# Supplementary material for: Complete Genome Sequencing Revealed the Potential Application of a Novel Weizmannia coagulans PL-W Production with Promising Bacteriocins in Food Preservative
Source: Foods. 2023 Jan 3;12(1):216. doi: 10.3390/foods12010216 (PMC9818457; doi:10.3390/foods12010216)
Supplement: Supplementary file 1 [file foods-12-00216-s001.zip › foods-1985011-supplementary.pdf]

Supplementary

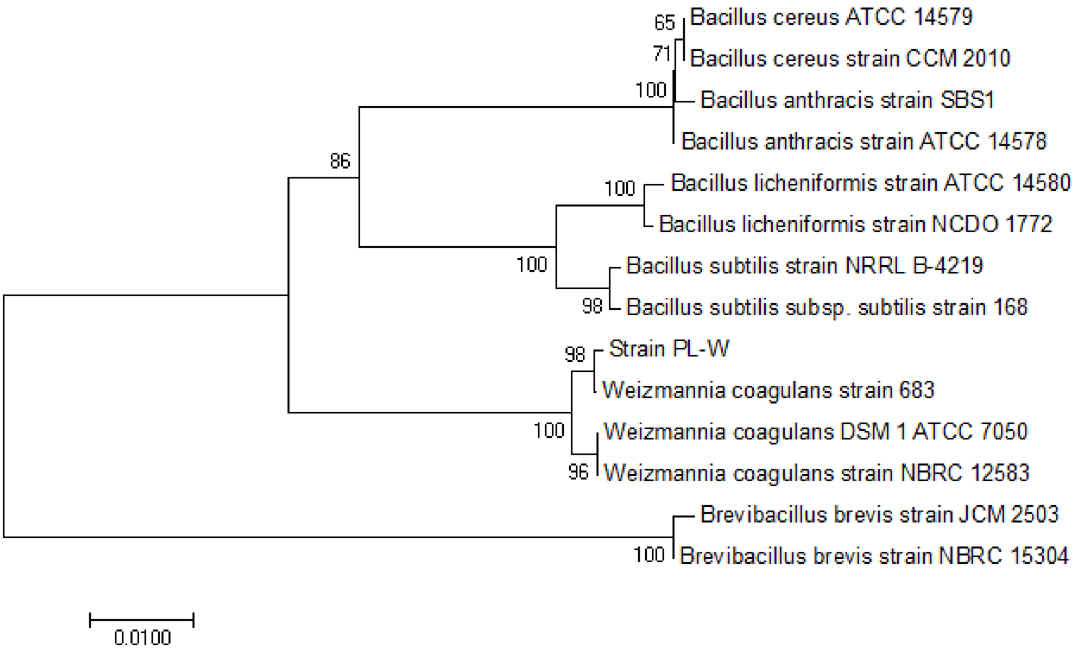

Figure S1. Phylogenetic analysis of 16S rRNA sequence of *W. coagulans* PL-W.

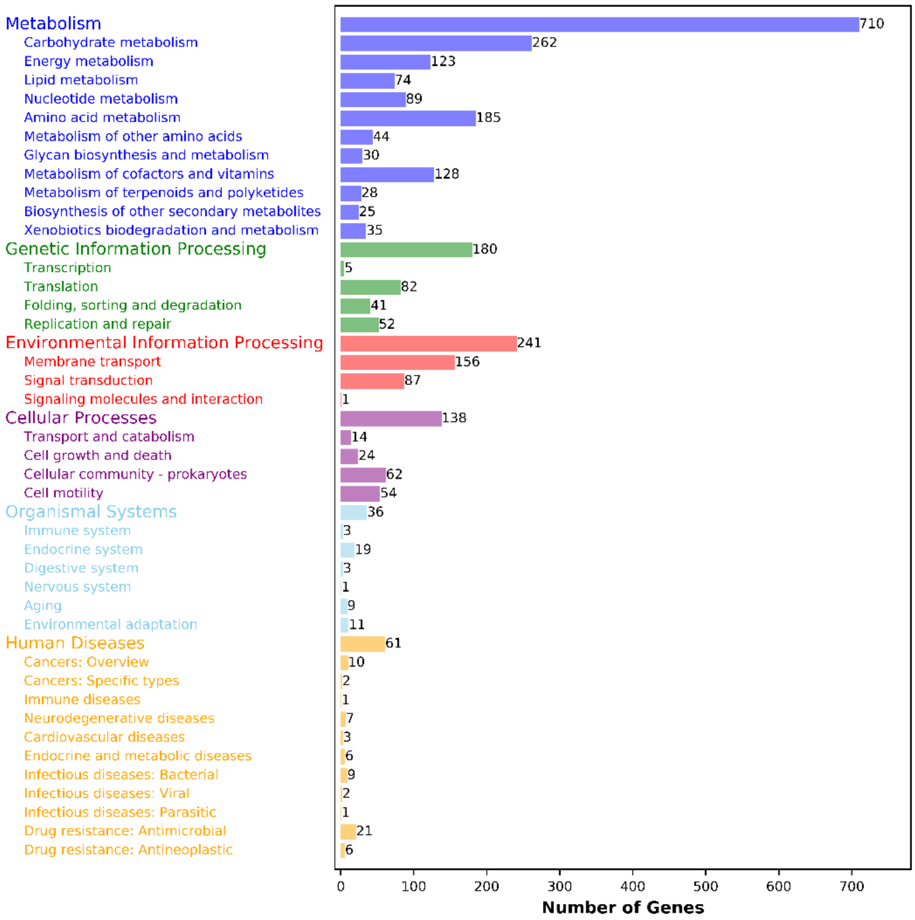

Figure S2. KEGG category distribution of functional annotation results.

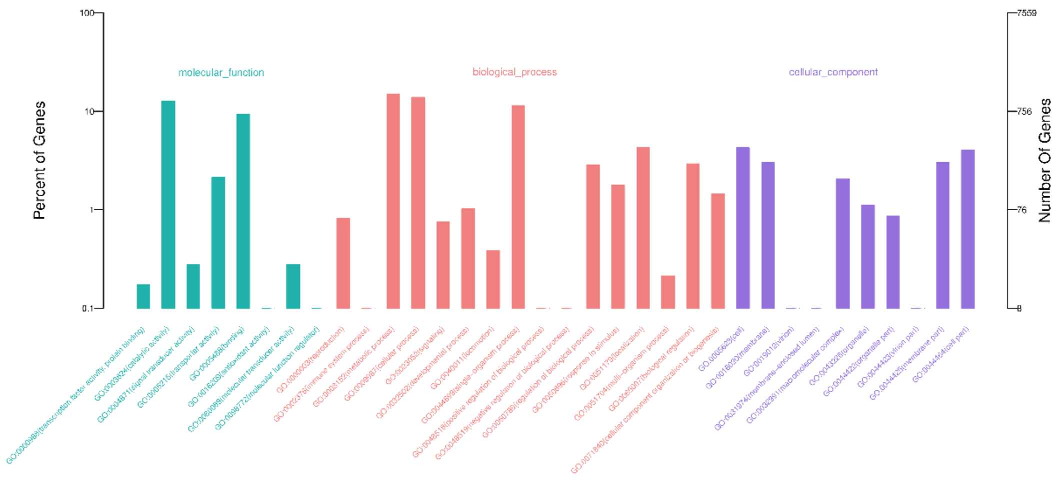

Figure S3. WEGO category distribution of functional annotation results.

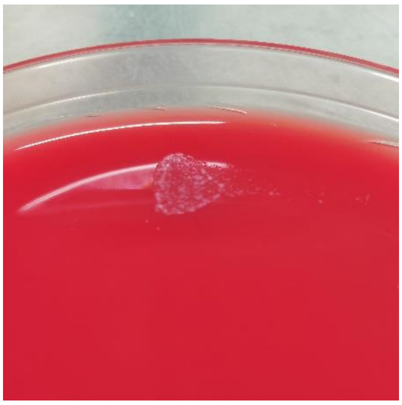

Figure S4. Evaluation of hemolytic activity of *W. coagulans* PL-W in Columbia blood agar plates.
